# Feature selection and semi-supervised clustering using multiobjective optimization

**DOI:** 10.1186/2193-1801-3-465

**Published:** 2014-08-26

**Authors:** Sriparna Saha, Asif Ekbal, Abhay Kumar Alok, Rachamadugu Spandana

**Affiliations:** Department of Computer Science and Engineering, Indian Institute of Technology Patna, Patna, India

**Keywords:** Clustering, Multiobjective optimization (MOO), Symmetry, Cluster validity indices, Semi-supervised clustering, Feature selection, Multi-center, Automatic determination of number of clusters

## Abstract

In this paper we have coupled feature selection problem with semi-supervised clustering. Semi-supervised clustering utilizes the information of unsupervised and supervised learning in order to overcome the problems related to them. But in general all the features present in the data set may not be important for clustering purpose. Thus appropriate selection of features from the set of all features is very much relevant from clustering point of view. In this paper we have solved the problem of automatic feature selection and semi-supervised clustering using multiobjective optimization. A recently created simulated annealing based multiobjective optimization technique titled archived multiobjective simulated annealing (AMOSA) is used as the underlying optimization technique. Here features and cluster centers are encoded in the form of a string. We assume that for each data set for 10% data points class level information are known to us. Two internal cluster validity indices reflecting different data properties, an external cluster validity index measuring the similarity between the obtained partitioning and the true labelling for 10% data points and a measure counting the number of features present in a particular string are optimized using the search capability of AMOSA. AMOSA is utilized to detect the appropriate subset of features, appropriate number of clusters as well as the appropriate partitioning from any given data set. The effectiveness of the proposed semi-supervised feature selection technique as compared to the existing techniques is shown for seven real-life data sets of varying complexities.

## 1 Introduction

Clustering, also termed as unsupervised learning, is the method of grouping the data items into different partitions or clusters in such a way so that points which belong to same cluster should be similar in some manner and points which belong to different clusters should be dissimilar in the same manner (Saha and Bandyopadhyay 2010). In supervised learning some training set needs to be available which captures the prior knowledge about class labels of some points. A classifier can be trained using this training set. After this step, a classifier can easily detect the class labels of unlabelled data depending on the model built. But in unsupervised classification, no prior knowledge about data points are available. Unsupervised learning classifies the data based on actual distribution of the data items and well quantified intrinsic property. In real-life it is easy to generate plenty of unlabeled data but it is hard to determine actual annotations of these data. Some human annotators are required to annotate the data. Thus training set generation is very much cost expensive and requires more time. Because of this situation unsupervised learning methods are widely used than supervised learning but the accuracies obtained by unsupervised clustering techniques are lower than supervised classification techniques. Thus it is required to develop some new classification techniques which can combine the fruitfulness of both supervised and unsupervised classification techniques. This new classification technique is named as semi-supervised classification and it is developed to solve the problems associated with unsupervised and supervised classification methods. Both supervised and unsupervised classification information are used in semi-supervised classification. Because of the above mentioned property, semi-supervised classification is more demanding and showing good results. This has a large number of applications in natural Language processing, information retrieval, data mining, gene function classification, protein classification and bioinformatics (Handl and Knowles 2006; Li et al. 2003). In this paper we have solved some problems related to semi-supervised clustering techniques.

Feature selection, or subset selection, is the method of reducing dimensionality in machine learning. It is important for different reasons: first total computation can be reduced if we can reduce the dimensionality. Secondly all the features may not be helpful to classify the data; some may be redundant and irrelevant from the classification point of view. Thus it is needed to determine automatically relevant subset of features. In order to address the above mentioned problems, feature selection is needed both for unsupervised as well as supervised classification problems. There are many works to address the feature selection problem in supervised domain (Aha and Bankert 1996; Bermejo et al. 2012; Blum and Langley 1997; Liu and Yu 2005). But there are a very few works for solving the feature selection problems from unsupervised domain. In case of unsupervised classification it is very difficult to measure the goodness of a particular feature. In recent years some works have been reported to solve the unsupervised feature selection problem (Dash and Liu 1999; Dy and Brodley 2004; Kim et al. 2002; Mitra et al. 2002; Peña et al. 2001; Talavera 1999). But most of these techniques pose the feature selection problem as a single objective optimization technique. They have mostly optimized a single cluster quality measure. In recent years there are some approaches which use multiobjective optimization to solve the unsupervised feature selection problem. A multiobjective wrapper based approach to solve the unsupervised feature selection problem is developed by Morita et al. (2003). *K*-means clustering technique is utilized as the underlying partitioning method and authors have varied the number of clusters in a range. They have utilized a multiobjective evolutionary algorithm (the Non-dominated Sorting GA-II, NSGA-II, (Deb et al. 2002)) as the underlying optimization tool. Two objective functions related to clustering are deployed for the clustering task namely the number of features and the Davies-Bouldin-Index (DB-Index, Davies and Bouldin (1979)). In 2002, Kim et al. (2002) presented a multiobjective approach for wrapper based unsupervised feature selection. A multiobjective algorithm ELSA (Evolutionary Local Selection Algorithm (Menczer et al. 2000)) is utilized as the underlying optimization technique. Authors have used *K*-means algorithm as the underlying partitioning tool to partition the given data set based on a feature combination. Both good feature subsets and the corresponding numbers of clusters are determined by ELSA. Authors have considered four different clustering objectives for optimization: a count on number of features, the number of clusters present in a chromosome, intra-cluster compactness of the partitioning encoded in a chromosome and inter-cluster separation of the partitioning encoded in a chromosome. The problem of feature selection coupled with clustering is also treated as a multiobjective optimization problem in (Julia and Knowles 2006). There a multiobjective evolutionary algorithm named Pareto envelope-based selection algorithm version 2 (PESA-II) is used to develop a multiobjective feature selection technique. But this technique utilizes Euclidean distance for assigning points to different clusters. This technique utilizes K-means as the underlying clustering technique. Thus it can only be able to determine hyperspherical shaped equal sized clusters. In this paper we have posed the problem of feature selection for semi-supervised classification as a multiobjective optimization problem. A recently created simulated annealing based multiobjective optimization technique, AMOSA (Bandyopadhyay et al. 2007), is utilized as the underlying optimization tool. Instead of using Euclidean distance, Point symmetry based distance (Bandyopadhyay and Saha 2007) is utilized for assigning points to different clusters.

The proposed multiobjective semi-supervised clustering as well as feature selection technique called Semi-FeaClusMOO technique encodes number of features and number of cluster centers in the form of a string. Then points are assigned based on the features present in the string to different clusters using a recently introduced point symmetry based distance (Bandyopadhyay and Saha 2007). As supervised information, we assumed that class labels of 10% data points are available to us. Four different objective functions are used for optimization. These are a symmetry based cluster validity index, *Sym*-index (Bandyopadhyay and Saha 2007), Euclidean distance based cluster validity index, XB-index (Xie and Beni 1991), an external cluster validity index, *Adjusted Rand Index* (Yeung and Ruzzo 2001) which measures the similarity of the obtained partitioning on labeled data points with their original class labels, and number of features. The third objective function captures the supervised information. The fourth objective function is used to balance the bias of the first two objective functions on dimensionality. Some distance computations have to be performed in order to determine the values of internal cluster validity indices. As value of distance function decreases with the decrease of number of features, internal cluster validity indices are biased towards lower dimensions (Julia and Knowles 2006). In order to balance these bias we have used the fourth objective which will try to increase the number of features present in a data set. The final Pareto optimal front contains a set of solutions representing different feature combinations and cluster centers. The algorithm will automatically identify the appropriate number of clusters, appropriate partitioning and automatic feature combinations from a data set. Results are shown for several higher dimensional real-life data sets. The performance of Semi-FeaClusMOO technique is compared with a) Semi-FeaClusMOO technique using Euclidean distance in place of point symmetry based distance for assigning points to different clusters b) a multiobjective based simultaneous feature selection and unsupervised clustering technique where only three objective functions are used: *Sym*-index, *XB*-index and number of features. Same architectures of AMOSA based clustering technique proposed in this paper are followed; (c) a multiobjective based automatic clustering technique where all the features are utilized for distance computation and point symmetry based distance is used for assigning points to different clusters, (d) a single objective based automatic clustering technique where all the features are used for distance computation and point symmetry based distance is used for assigning points to different clusters and e) *K*-means clustering technique with all feature combinations and known number of clusters.

## 2 The SA based MOO algorithm: AMOSA

Archived multiobjective simulated annealing (AMOSA) (Bandyopadhyay et al. 2007) is a newly developed simulated annealing (SA) based multiobjective optimization technique. MOO is utilized for solving different real-world problems having multiple objective functions to be optimized simultaneously. Simulated annealing (SA), based on the principles of statistical mechanics (Kirkpatrik et al. 1983), is a search tool in order to solve difficult problems related to optimization theory. SA dominates over exhaustive search procedures because of its time and resource efficiencies. SAs are widely used to solve single objective optimization problems. But there are very few attempts in solving multiple objective optimization problems using SAs. This is because of search-from-a-point nature of SA which helps it to determine a single solution after a single run. In case of multi-objective optimization, there are a set of trade-off solutions. In order to generate all the solutions, we need to run SA multiple times. In recent years, Bandyopadhyay et al. developed an efficient multiobjective version of SA called AMOSA (Bandyopadhyay et al. 2007) which overcomes all the above stated limitations.

Several new concepts have been introduced in AMOSA (archived multiobjective simulated annealing) (Bandyopadhyay et al. 2007) which is an multiobjective version of SA. The concept of an archive is utilized in AMOSA which is used to store all the non-dominated solutions seen so far. This archive is associated with two limits: a hard or strict limit denoted by *HL*, and a larger, soft limit denoted by *SL*, where *SL*>*HL*. During the SA process several non-dominated solutions are generated which all are stored in the archive. In the mean while if a newly generated solution dominates some members of the archive, then those solutions are removed from the archive. During the process, if the size of the archive exceeds the specified soft limit, *SL*, clustering procedure is invoked to reduce the size to *HL*.

The AMOSA algorithm starts its execution with the initialization of a number (*γ*×*SL*,*γ*>1) of solutions in the archive, each of which represents a state in the search space. For each solution, multiple objective functions are computed. Each of these newly generated solutions is then fine-tuned by using simple hill-climbing and domination relation for a number of iterations. Thereafter non-domination sorting is applied and all the non-dominated solutions are stored in the archive until the size of the archive crosses the soft limit *SL*. If archive crosses *HL* after this initialization process, single linkage clustering procedure is called in order to reduce the size of the archive to *HL*. A point from the archive is randomly picked up. At temperature, *T*=*Tmax*, this is considered as the current-pt, or the initial solution. The current-pt is mutated to produce a new solution called the new-pt. The objective functional values of new-pt are calculated. new-pt is now compared with current-pt and points in the archive. A new term called amount of domination, *Δdom*(*a*,*b*) between two solutions a and b is introduced in AMOSA and it is defined as follows:, where *f*_*i*_(*a*) and *f*_*i*_(*b*) are the *i*th objective values of the two solutions and *R*_*i*_ is the corresponding range of the objective function. After comparing the domination status of the new-pt, current-pt and points in the archive, different cases may arise viz., accept the (i) new-pt, (ii) current-pt, or, (iii) a solution from the archive. During the process if archive size crosses *SL*, clustering is again invoked to reduce the size to *HL*. The above mentioned steps are executed *iter* times for each temperature which is annealed with a particular cooling rate of *α*(<1) until the minimum temperature *Tmin* is attained. Finally the procedure is completed with the final archive containing the final non-dominated solutions.

Results show that performance of AMOSA is better than some other well-known MOO algorithms especially for 3 or more objectives (Bandyopadhyay et al. 2007). Inspired by these results AMOSA is utilized in the current paper as the underlying MOO technique.

## 3 Proposed method of multiobjective feature selection and semi-supervised clustering technique

This section describes the newly proposed multiobjective feature selection and semi-supervised clustering technique, *Semi-FeaClusMOO*, in detail.

### 3.1 String representation and population initialization

**Figure 1:**
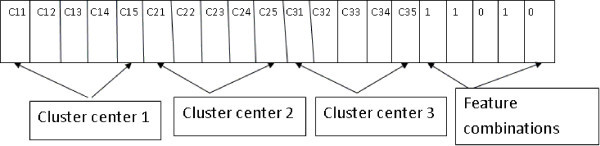
**Example of representing cluster centers and features in the form of a string.**

In *Semi-FeaClusMOO*, a state of AMOSA is comprising of two items: a) a set of real numbers which represents the coordinates of the centers of the clusters hence the associated partitioning of the data and b) a set of binary numbers which represents different feature combinations. AMOSA tries to determine an appropriate set of cluster centers and the appropriate set of feature combinations. Suppose a particular string encodes the centers of *K* number of clusters and total number of available features is denoted by *F*. Then the length of the string will be *F*+*K*×*F*. The *K* number of cluster centers are randomly chosen *K* points from the data set. Here feature combinations are some randomly chosen binary numbers. An example of a string is given below as well as in Figure 1 where K =3 and . This represents a partitioning having three cluster centers , , and  and we have to only consider the features: first, second and fourth. These features are considered for cluster assignments and objective function calculations.

Initially *K*_*i*_ number of clusters are encoded in each string, such that *K*_*i*_=(*rand*()mod(*K*^*max*^-1))+2. Here, *rand*() is a random number generator returning an integer, and *K*^*max*^ is the soft estimate of upper limit of number of clusters. For a particular string number of clusters encoded in it can vary between 2 to *K*^*max*^.

For the initialization purpose, we have followed a random procedure. For a particular string *i* total number of cluster centers encoded in it is *K*_*i*_. These *K*_*i*_ number of cluster centers are selected randomly from the entire data set. Thereafter these cluster centers are encoded in that particular string. Initial paritioning is generated randomly using minimum center distance based criterion. The feature part of each string is also initialized randomly. Suppose in the data set there are *F* number of features; then each position of the feature set is randomly initialized to either 0 or 1. Here value of 0 at the *i*th position represents that this feature will not take part in further processing and value of 1 denotes that *i*th feature will participate in further processing like cluster assignment, objective value computations etc.

### 3.2 Assignment of points

For assignment of points to different clusters, the newly developed point symmetry based distance (Bandyopadhyay and Saha 2007), , is utilized. This distance is developed in (Bandyopadhyay and Saha 2007). The below description is taken from (Bandyopadhyay and Saha 2007). This distance is computed as follows. *Suppose a point is denoted by**. The symmetrical (reflected) point of**with respect to a particular center**is**. Let us denote this by**. Let knear unique nearest neighbors of**be at Euclidean distances of d*_*i*_*s, i*=1,2,…*knear. Then*12

*where**is the Euclidean distance between the point**and**, and**is a symmetry measure of**with respect to**and is defined as**. Here knear is chosen equal to 2. The properties of**are thoroughly described in (*Bandyopadhyay and Saha 2008*).*

In order to assign points to different clusters, each center encoded in a string is considered as a separate cluster. Suppose *K* number of clusters and *f* number of features are encoded in a particular string. Here cluster *k* is selected for assignment of point , 1≤*i*≤*n*, iff , and . For , point  is assigned to some cluster *m* iff . In other words, point  is assigned to that particular cluster with respect to whose center its point symmetry based distance, *d*_*ps*_ (Bandyopadhyay and Saha 2007), is minimum, provided the corresponding *d*_*sym*_ value is less than some threshold *θ*. Otherwise, cluster assignment is based on minimum Euclidean distance criterion as normally done in (Bandyopadhyay and Maulik 2002) or the *K*-means algorithm. The possible reasons for assignment of points to different clusters using the above mentioned way are as follows: In the intermediate stages of the algorithm, when the centers are not set to the proper values, the minimum *d*_*ps*_ value for a point is expected to be very large as the point might not be symmetric with respect to any center. In such cases, we have used Euclidean distance for cluster assignment. In contrast, when cluster centers are set to the appropriate values, *d*_*ps*_ values become reasonably small and cluster assignment based on point symmetry based distance is more meaningful. For the point symmetry based distance computation, *d*_*ps*_ (Bandyopadhyay and Saha 2007) and Euclidean distance computation we have considered only those features which are present in that particular string.

As explained in Ref (Bandyopadhyay and Saha 2007), the value of *θ* is set equal to the maximum nearest neighbor distance among all the points in the data set.

### 3.3 Objective functions used

For the purpose of optimization, three different cluster validity indices and another objective function counting the number of features present in the data set are considered. The first two objective functions are two internal cluster validity indices which reflect different properties of good clustering solutions. The first measures the amount of symmetry present in a particular partitioning. The second one measures the goodness of the clusters in terms of the Euclidean distance. The third cluster validity index is an external cluster validity index named *Adjusted Rand Index* (Yeung and Ruzzo 2001). The fourth objective function measures the number of features present in a particular string.

The first objective function is the point symmetry distance based cluster validity index, *Sym*-index (Bandyopadhyay and Saha 2008). This validity index quantifies the goodness of a partitioning in terms of symmetricity. It is able to identify symmetrical shaped well separated clusters. Second cluster validity index, XB-index (Xie and Beni 1991), is based on Euclidean distance. It is able to identify compact well-separated clusters. Here compactness is measured in terms of Euclidean distance. In order to compute the *Sym*-index and XB-index values corresponding to a particular string we have utilized those features which are present in that particular string.

In order to compute the similarity between the obtained partitioning and true class labels of the 10% data points for which original class label information are known, we have used an external cluster validity index as the third objective function. The use of this index provides a way of incorporating the labeled information in the unsupervised clustering. As the external cluster validity index, we have used Adjusted rand index (ARI) (Yeung and Ruzzo 2001). This is the third objective function which captures the matching between observed solution and prior knowledge based true solution for the 10% data points. ARI is computed over only these 10% data points for which class information are known. When two partitions agree completely then adjusted rand index value is 1. Thus higher values of adjusted rand index are better.

The fourth objective function is the number of features encoded in a particular string. We have to calculate the number of features present in a particular string and have to maximize the value of number of features. *f*_3_=maximize∥*f*∥ where ∥*f*∥=number of features present in that particular string.

The above four cluster validity indices are computed for each string. For *Sym*-index and XB-index computations we have to consider only those features which are present in that particular string. ARI index value is calculated only for 10% data for which labeled information are known. Thus the objective functions for a particular string are as follows:


where *Sym*(*K*,*f*), *XB*(*K*,*f*), *Adj*(*K*), and ∥*f*∥ denote respectively, the calculated *Sym*-index value, *XB*-index value, *Adjusted Rand Index* value and number of features present in that particular string. Here number of clusters present in a particular string is denoted by *K* and *f* is the number of features present in that particular string. The search capability of newly developed simulated annealing based MOO algorithm, AMOSA is utilized to maximize simultaneously above mentioned four objective functions.

### 3.4 Mutation operation

Here mutation operations are applied on a particular string to generate a new string. In order to change the cluster centers encoded in a particular string three different types of mutation operations are applied. Binary mutation is applied to change the feature combinations present in a particular string. Here each bit position of the feature vector is flipped with some probability (if initially there was 1- it is now replaced by 0; if initially there was 0- it is now replaced by 1).

1. In order to mutate each individual cluster center, we have used Laplacian distribution, , where the scaling factor *δ* is used to set the magnitude of mutation to generate a new value for that particular position. Here *μ* represents the value at the position which is to be mutated. We have kept scaling factor *δ* equals to 1.0. The newly generated value is used to replace the old value. This perturbation operation is applied to all dimensions independently. In order to change the feature combinations binary mutation is applied.
2. In order to reduce the number of clusters encoded in a string by 1, a cluster center is removed from the string. In this case again to change the feature combinations binary mutation is used.
3. In order to increase the number of clusters encoded in a string by 1, we have to add an arbitrarily chosen data point in the string. Here the cluster center to be added is a randomly chosen data point from the entire data set. In this case again to change the feature combinations binary mutation is used.

For a particular string, if it is chosen for mutation, any of the above defined types of mutation operation is applied with uniform probability.

### 3.5 Selection of the best solution

After application of any MOO based technique we used to get a set of non-dominated solutions (Deb 2001) on the final Pareto optimal front. Each of these solutions provides some feature combinations and cluster centers. Based on these information using the point symmetry based distance (Bandyopadhyay and Saha 2007) we can get the partitioning associated with this solution. All the solutions are equally important from execution point of view. But sometimes depending on user requirement we may need to select a single solution. In this paper we have used a semi-supervised approach to select a single solution.

In the proposed semi-supervised feature selection algorithm we have assumed that class labels of 10% data points are known to us. The proposed Semi-FeaClustMOO generates a set of Pareto optimal solutions on the final archive. Based on the cluster centers and feature combinations present in these solutions we used to assign the cluster labels of these 10% labeled points using the nearest center criterion. The similarity score of these assigned class labels and the original class labels is measured using an external cluster validity index, named *Minkowski Score* (Jiang et al. 2004). *Minkowski Score* quantifies the quality of a solution provided the true clustering (Jiang et al. 2004). Suppose T denotes the “true” clustering solution and S denotes the partitioning result which we wish to measure. Let the number of pairs of elements that are in the same cluster in both S and T is denoted by *n*_11_, the number of pairs that are in the same cluster only in S is denoted by *n*_01_, and the number of pairs that are in the same cluster in T but in different clusters in S is denoted by *n*_10_. *Minkowski Score* is then calculated as below:
3

Here optimum value is 0. Lower values of MS are preferred.

The solution which attains the minimum *Minkowski Score* value calculated over the labeled points is selected as the best solution.

## 4 Experimental results

In this section we have discussed about the results obtained after application of Semi-FeaClusMOO technique on some real-life data sets.

### 4.1 Data sets used

Seven real-life data sets obtained from http://www.ics.uci.edu/~mlearn/MLRepository.html are used for the experiments. The detailed description of the real-life data sets in terms of the total number of points present, dimension of the data set and the number of clusters are presented in Table 1.

**Table 1:** **Description of data sets**

Data set	# points	Dimension (d)	Actual number
			of clusters (K)
*Iris*	150	4	3
*Cancer*	683	9	2
*Newthy.*	215	5	3
*Wine*	178	13	3
*LiverDis.*	345	6	2
*LungCan.*	33	56	2
*Glass*	214	9	6

1. *Iris*: This data set is having 150 data points distributed over 3 clusters. Here each cluster is having 50 points. This data set corresponds to different types of irises having four feature values (Fisher 1936). There are three classes in the data set. These are Setosa, Versicolor and Virginica. Among these three classes, two classes (Versicolor and Virginica) are overlapping to each other, while the third one is linearly separable from the other two.
2. *Cancer*: This data set represents Wisconsin Breast *Cancer* data set. There are total 683 sample points, each having nine features. The features correspond to clump thickness, cell size uniformity, cell shape uniformity, marginal adhesion, single epithelial cell size, bare nuclei, bland chromatin, normal nucleoli and mitoses. The data set is having two classes : malignant and benign. These two classes are known to be well separated from each other.
3. *Newthyroid*: This data set corresponds to Thyroid gland data. There are total three classes present in the data. These are: euthyroidism, hypothyroidism and hyperthyroidism. Each sample represents values of five laboratory tests which are conducted to predict whether a patient’s thyroid belongs to any of the three classes. This data set is having total 215 samples.
4. *Wine*: This data set corresponds to Wine recognition data. It is having total 178 instances, each is having 13 features. These features correspond to different chemical analysis of wines. The samples are grown in the same region in Italy but derived from three different families. There are three classes present in the data set. The Chemical analysis determines the magnitudes of 13 constituents found in each of the three types of wines.
5. *LiverDisorder*: This data set corresponds to liver disorder data. There are total 345 instances in the data set. Each instance is having 6 features. There are two output classes for this data set.
6. *LungCancer*: This data is having 32 samples; each having 56 features. This data represents different samples of pathological lung cancers where there are three different categories.
7. *Glass*: This data corresponds to glass identification data. It is consisting of 214 samples where each sample is having 9 features (an Id# feature has been removed). From the criminological investigation point of view, this study of the classification of the types of glass is important. At the scene of the crime, the broken glasses can be utilized as some evidences if these are identified correctly. The data set is having total 6 classes present.

## 5 Discussion of results

In Semi-FeaClusMOO, the newly developed simulated annealing based MOO technique, AMOSA, is used as the underlying optimization technique for simultaneous feature selection and semi-supervised clustering. The parameters of the proposed Semi-FeaClusMOO clustering technique are as follows: *SL* = 100 *HL* = 50, *iter* = 50, *Tmax* = 100, *Tmin* = 0.00001 and cooling rate, *α*=0.9. The performance of Semi-FeaClusMOO technique is compared with a) Semi-FeaClusMOO using Euclidean distance in place of point symmetry based distance for assignment of points to different clusters b) FeaClusMOO: a multiobjective based simultaneous feature selection and clustering technique which uses AMOSA as the underlying optimization strategy. Here three objective functions are optimized: *Sym*-index, *XB*-index and number of features. No external cluster validity index is considered. (c) VAMOSA (Saha and Bandyopadhyay 2010): a point symmetry based multiobjective automatic clustering technique which can tackle only the clustering problem using multiobjective optimization. Here two cluster validity indices are considered for optimization: *Sym*-index and *XB*-index. Here all the features are utilized for distance computation and point symmetry based distance is used for assignment of points to different clusters. (d) a point symmetry based automatic clustering technique utilizing the search capability of GAs, VGAPS-clustering (Bandyopadhyay and Saha 2008) where all the features are utilized for distance computation and point symmetry based distance is utilized for assignment of points to different clusters c) traditional *K*-means clustering technique with all the features utilized for distance computation. Table 2 reports the number of clusters and the number of features automatically determined by the proposed Semi-FeaClusMOO technique using point symmetry based distance, Semi-FeaClusMOO technique using Euclidean distance in place of point symmetry based distance, FeaClusMOO technique for feature selection and unsupervised clustering, VAMOSA technique for only unsupervised clustering, and VGAPS clustering technique (Bandyopadhyay and Saha 2008) for all the above mentioned data sets. The *Minkowski Score* values of the final clusterings identified by these six algorithms are also shown in Table 2. *Minkowski Score* is an external cluster validity index which measures the goodness of an obtained partitioning. Comparison of our proposed technique is also carried out with a traditional clustering technique, *K*-means. This algorithm is executed on all data sets with actual number of clusters and with all the available features. The final *Minkowski Score* values are reported in Table 2.

**Table 2:** **Results on different data sets by different algorithms**

Data set	Semi-FeaClusMOO			Semi-FeaClusMOO _***Euc***_			FeaClusMOO			VAMOSA		VGAPS		KM
	Fea	OC	MS	Fea	OC	MS	Fea	OC	MS	OC	MS	OC	MS	MS
*Iris*	4	3	0.39	2,3,4	4	0.40	3,4	3	0.44	2	0.80	3	0.62	0.68
*Cancer*	1,2,3,5,6	2	0.31	1,2,5,6,7	2	0.37	1,2,3,5,6	2	0.31	2	0.32	2	0.37	0.37
*Newthy.*	2,4	3	0.46	2-4	3	0.47	1,2,4,5	3	0.54	5	0.57	3	0.58	0.94
*Wine*	1,3,6,8,9,12	3	0.62	1,6,7,10,12	6	0.64	1,6,7	3	0.67	3	0.97	3	1.12	1.40
*LiverDis.*	1,6,7,10,12	2	0.64	1,2,3,6	3	0.98	1,2,5	2	0.98	2	0.98	2	0.98	0.98
*LungCan.*	1-4,	2	0.70	2-4	4	0.71	1-4,	2	0.70	3	0.85	3	1.24	1.45
	7-8,10,			6-9			7-8,10,							
	11,13,16			11-14			11,13,16							
	19-23,			16,22			19-23,							
	25-27			27-32			25-27							
	29-31			34,36-38			29-31							
	32-39			41,43-46			32-39							
	42-45			48,49			42-45							
	47-49			51-56			47-49							
	51-53						51-53							
	55-56						55-56							
*Glass*	1-4,7-9	6	1.03	4,9	10	1.05	1,2,3,4,5	6	1.05	6	1.08	6	1.10	1.69

Here *d*, *K*, *Fea*, *OC*, *MS* denote respectively the original dimension, the number of features selected by the algorithm, the number of clusters originally present, obtained number of clusters and *Minkowski Score*, respectively. Each algorithm is executed ten times and the best results among these ten runs are reported.

In Semi-FeaClusMOO _*Sym*_, Semi-FeaClusMOO _*Euc*_, FeaClusMOO technique, VAMOSA technique, AMOSA is used as the base optimization tool. Thus same parameter setting is utilized in each of these cases. The parameters are as follows: *SL* = 100 *HL* = 50, *iter* = 50, *Tmax* = 100, *Tmin* = 0.00001 and cooling rate, *α*=0.9.

The parameter values of VGAPS clustering are kept as follows: population size is set to 100, number of generations is set to 60 (if the algorithm is executed for more number of generations, no performance improvement is observed). Adaptive mutation and crossover probabilities are used in case of VGAPS. Note that we have set the parameters in such a way that all the algorithms are executed for equal number of function evaluations. Total function evaluations computed by AMOSA based approaches are equal to the total function evaluations computed by VGAPS and *K*-means clustering techniques. Here each of the above mentioned algorithms are applied ten times on each data set and Table 2 reports the best results out of these ten different runs.

In order show the efficacy of the proposed feature selection and clustering technique, Semi-FeaClusMOO clustering, we have used several real-life data sets. These real-life data sets are higher dimensional in nature; thus these are suitable for the application of some feature selection technique. For these real-life data sets, we can not demonstrate the results visually as these are higher dimensional data sets. Here we have reported the best *Minkowski Scores* obtained by all the algorithms over 10 different executions for all data sets. For *Iris* data set Semi-FeaClusMOO clustering technique selects only a single feature out of total four features. It is able to identify the appropriate partitioning (*K*=3) with this single feature. The *Minkowski Score* value of this partitioning is the lowest compared to other five techniques (refer to Table 2). Semi-FeaClusMOO with Euclidean distance selects three features out of total four features on its final solution. It is not also able to correctly identify appropriate number of clusters. The corresponding MS value is larger than that obtained by Semi-FeaClusMOO with point symmetry based distance. This shows the utility of point symmetry based distance for assigning points to different clusters. FeaClusMOO technique is also able to determine the appropriate number of clusters. It identifies two features out of total 4 features. But the corresponding MS value is higher compared to the proposed feature selection as well as semi-supervised clustering technique. VAMOSA and VGAPS-clustering are applied with all features on the given data set. They attain the MS values of 0.80 and 0.62, respectively. While VGAPS is able to identify appropriate number of clusters from this data set automatically, VAMOSA fails to do so. The MS values obtained by VAMOSA and VGAPS clustering techniques are, respectively, 0.23 and 0.41 points higher than that attained by the proposed Semi-FeaClusMOO clustering technique. As minimum MS value means better partitioning, it proves the utility of feature selection. *K*-means performs poorly for this data set. It attains MS value of 0.68. Cancer data set is having total nine features. Out of these nine features, Semi-FeaClusMOO technique selects total 5 features on the optimal solution. The corresponding partitioning is having two clusters with the minimum MS value (refer to Table 2). Semi-FeaClusMOO _*Euc*_ technique again selects 5 features on its optimal solution. It is able to identify the correct number of clusters from this data set. But the MS value obtained by this algorithm is higher than MS value obtained by Semi-FeaClusMOO technique. This again proves the utility of assigning points to different clusters based on the point symmetry based distance. FeaClusMOO technique performs similarly as Semi-FeaClusMOO. It also attains a minimum MS value of 0.31. It selects total five features and two clusters on the optimal partitioning. This result proves that for this data set no improvement is observed after utilizing the labeled information (refer to Table 2). VAMOSA and VGAPS clustering techniques are applied on this data set with all the features. Both the algorithms identify correct number of clusters from this data set. VAMOSA attains MS value of 0.32 which is slightly higher than the MS value obtained by Semi-FeaClusMOO technique (refer to Table 2). The MS values obtained by VGAPS clustering and *K*-means clustering are the same (refer to Table 2).

For Newthyroid data, proposed Semi-FeaClusMOO clustering technique selects 2 features out of total 5 features on its final solution. It is able to identify the appropriate number of clusters from this data set. The MS value attained by this clustering technique is also the minimum (refer to Table 2). Semi-FeaClusMOO _*Euc*_ identifies three features on its final solution. It is also able to identify the correct number of clusters. The MS value attained by this algorithm is slightly higher than the MS value obtained by Semi-FeaClusMOO clustering technique. FeaClusMOO clustering technique selects 4 features out of total 5 features. But it is also able to identify the appropriate number of clusters from this data set. The corresponding MS value is higher compared to that obtained by the proposed Semi-FeaClusMOO clustering technique (refer to Table 2). This proves the utility of using semi-supervised clustering technique. VAMOSA clustering technique selects total 5 clusters from this data set when applied with all the available features. The corresponding MS score is 0.57 which is 0.11 points higher than that obtained by Semi-FeaClusMOO technique. VGAPS clustering technique with all the features again also identifies the correct number of clusters for this data set. But the MS value obtained by this clustering technique is higher compared to that obtained by Semi-FeaClusMOO clustering technique. *K*-means clustering technique performs poorly for this data set. Results on this data set again proves the utilization of feature selection.

For wine data, proposed Semi-FeaClusMOO clustering technique is able to detect proper number of clusters (*K*=3). It selects total 6 features out of 13 features. The corresponding MS value is also the lowest among all the clustering techniques (refer to Table 2). Semi-FeaClusMOO _*Euc*_ clustering technique with Euclidean distance is not able to detect the appropriate number of clusters from this data set. It identifies total 5 clusters from this data set. The MS value attained by this technique is slightly higher than that obtained by Semi-FeaClusMOO technique. This again proves the utility of using point symmetry based distance. FeaClusMOO technique identifies three features on the final solution. It is able to correctly detect the number of clusters from this data set. But the MS score obtained by this algorithm is slightly higher than that obtained by Semi-FeaClusMOO clustering technique. This again shows the utility of using semi-supervised information. VAMOSA clustering technique when applied with all the features is able to detect the correct number of clusters but it attains a very high MS value. VGAPS clustering technique when applied with all the features is able to identify appropriate number of clusters. The corresponding MS value is 1.12 which is 0.50 points higher than MS score obtained by the proposed Semi-FeaClusMOO clustering technique. This shows the utility of feature selection. *K*-means clustering technique performs poorly for this data set. Results on this data set reveal that Euclidean distance based clustering techniques are not suitable for this data set.

For LiverDisorder data set, Semi-FeaClusMOO technique performs the best as compared to other techniques (refer to Table 2). It selects five features out of total six features and identifies appropriate number of clusters from this data set. The MS value obtained by this technique is the lowest among all. Semi-FeaClusMOO _*Euc*_ clustering technique selects four features on its final solution. It determines three clusters from the data set. The MS score attained by this technique is 0.34 times higher than MS value obtained by Semi-FeaClusMOO technique. This again proves the utility of using point symmetry based distance. FeaClusMOO also determines three features on the final solution. The final partitioning identified by this algorithm has *K*=2 number of clusters. The MS value attained by this technique is also much higher than that obtained by Semi-FeaClusMOO technique. This proves that the use of labeled information helps to improve the performance of the proposed semi-supervised technique. VAMOSA and VGAPS perform similarly for this data set. They have applied with all the available feature sets and identified similar MS scores. *K*-means also performs similarly.

For LungCancer data set, again the proposed clustering technique Semi-FeaClusMOO attains the minimum MS value (refer to Table 2). Appropriate number of clusters is also detected by this algorithm from this data set. FeaClusMOO technique performs similar to Semi-FeaClusMOO. Semi-FeaClusMOO clustering technique with Euclidean distance fails to identify appropriate number of clusters. VAMOSA, VGAPS and *K*-means clustering techniques fail for this particular data set. Results on this data set again reveal the utility of feature selection.

For Glass data set, proposed Semi-FeaClusMOO attains the minimum MS value (refer to Table 2). It is also able to identify appropriate number of clusters from this data set. It selects 7 features out of total 9 features. Semi-FeaClusMOO clustering technique with Euclidean distance fails to identify appropriate number of clusters. It identifies only two features out of total 9 features on the final solution. The MS value attained by Semi-FeaClusMOO clustering technique with Euclidean distance is slightly higher than that obtained by FeaClusMOO clustering technique with point symmetry based distance (refer to Table 2). FeaClusMOO technique identifies five features on the final solution. It correctly identifies appropriate number of clusters from the data set. But it attains some slightly higher values of MS score as compared to Semi-FeaClusMOO clustering technique. VAMOSA clustering technique when applied with all the available features on this data set attains MS value of 1.08 which is 0.05 points higher than Semi-FeaClusMOO clustering technique. It is able to identify correct number of clusters from this data set. VGAPS clustering technique when applied with all the available features on this data set is able to identify appropriate number of clusters. It also attains slightly higher values of MS score as compared to Semi-FeaClusMOO clustering technique. *K*-means clustering technique performs poorly for this data set. Results on this data set again reveal the utility of point symmetry based distance for data clustering.

### 5.1 Summary of results

Results on a wide variety of data sets show that the proposed feature selection and semi-supervised clustering technique Semi-FeaClusMOO is able to detect the appropriate feature combination, appropriate number of clusters and the appropriate partitioning from data sets having many different types of clusters. Use of point symmetry based distance enables the proposed algorithm to identify various symmetrical shaped clusters (hyperspheres, linear, ellipsoidal, ring shaped, etc.) having overlaps. Use of 10% labeled information helps Semi-FeaClusMOO to improve the performance of clustering. The proposed technique provides a way of incorporating some supervised knowledge in the unsupervised clustering problem. It combines two problems : feature selection and semi-supervised clustering. Results on real-life data sets show that Semi-FeaClusMOO is capable to detect partitioning from real-life data sets of varying characteristics. The results on seven real-life data sets establish the fact that Semi-FeaClusMOO is well-suited to detect clusters of widely varying characteristics. Results show that while Semi-FeaClusMOO with Euclidean distance is only able to detect hyperspherical shaped clusters well, VAMOSA and VGAPS are capable of doing so for symmetrical shaped clusters. FeaClusMOO technique does clustering and feature selection simultaneously. It is not capable of handling any supervised information. The proposed Semi-FeaClusMOO clustering technique is able to find out the proper clustering automatically where Semi-FeaClusMOO with Euclidean distance succeeds while FeaClusMOO/VAMOSA/VGAPS fails as well as where FeaClusMOO/VAMOSA/VGAPS succeeds while Semi-FeaClusMOO with Euclidean distance fails. Results also reveal the effectiveness of feature selection from the real-life data sets. The feature selection step of Semi-FeaClusMOO often helps it to perform better than VAMOSA/VGAPS clustering techniques which are also based on point symmetry based distance. Similarly use of labeled information helps Semi-FeaClusMOO to perform better than FeaClusMOO technique. Results show *K*-means in general fails to detect proper partitioning from real-life data sets.

The improved performance of Semi-FeaClusMOO can be attributed to the following facts. Use of labeled information helps to improve the clustering result. Use of point symmetry based distance helps it to detect clusters having symmetrical shapes. The symmetry based cluster validity index measures the total symmetry present in the obtained partitioning. The proposed algorithm is also capable of identifying the appropriate feature combinations. Finally, AMOSA, the underlying optimization technique makes it capable of optimizing four objective functions simultaneously.

### 5.2 Statistical test

We have executed some statistical tests guided by Demšar (2006) to prove the superiority of the proposed clustering technique, *Semi-FeaClusMOO*. Friedman statistical test (Friedman 1937) is conducted to check whether the six clustering techniques, Semi-FeaClusMOO, Semi-FeaClusMOO with Euclidean distance, FeaClusMOO, VAMOSA, VGAPS and *K*-means used here for experiment perform similarly or not. Some ranks are assigned by this test to each algorithm for each data set. It checks whether the calculated average ranks are significantly different from the average/mean rank. Friedman test on the above mentioned algorithms concludes that calculated average ranks and mean rank are different with a p value of 0.0166. The rankings of different algorithms are shown in Table 3. Thereafter we have conducted Nemenyi’s test (Nemenyi 1963) to compare the clustering techniques pairwise. For each of the cases, *α*=0.05 is kept. Results reveal that for all the cases, we have to reject the null hypotheses (the pairing algorithms perform similarly) as the corresponding *p* values are smaller than the *α*.

**Table 3:** **Computation of the rankings for the six algorithms considered in the study over 7 data sets, based on the** ***Minkowski Score*** **values obtained**

***Data set***	Semi-FeaClusMOO	Semi-FeaClusMOO _***Euc***_	FeaClusMOO	VAMOSA	VGAPS	KM
*Iris*	0.39(1)	0.39(1)	0.44(2)	0.80(5)	0.62(3)	0.68(4)
*Cancer*	0.31(1)	0.37(3)	0.31(1)	0.32(2)	0.37(3)	0.37(3)
*Newthyroid*	0.46(1)	0.47(2)	0.54(3)	0.57(4)	0.58(5)	0.94(6)
*Wine*	0.62(1)	0.64(2)	0.67(3)	0.97(4)	1.12(5)	1.40(6)
*LiverDisorder*	0.64(1)	0.98(2)	0.98(2)	0.98(2)	0.98(2)	0.98(2)
*LungCancer*	0.70(1)	0.71(2)	0.70(1)	0.85(3)	1.24(4)	1.45(5)
*Glass*	1.03(1)	1.05(2)	1.05(2)	1.08(3)	1.10(4)	1.69(5)
Average rank	1	2	2	3.28	3.71	6.2

## 6 Conclusion

In this paper the problem of simultaneous feature selection and semi-supervised clustering is formulated as a multiobjective optimization problem. Semi-supervised clustering is a new domain where concepts of supervised classification and unsupervised classification have been combined. It utilizes few amount of labeled data and a large amount of unlabeled data. In this paper we have solved the problem of semi-supervised clustering using multiobjective optimization. We have proposed a new way of utilizing the labeled information for solving the unsupervised classification problem. For clustering all the features of a data set may not be important. Thus we have firstly selected some features and then performed semi-supervised clustering based on these features. A new multiobjective (MO) clustering technique Semi-FeaClusMOO is proposed which can automatically (a) identify appropriate set of features from the data set, (b) partition the data into an appropriate number of clusters and (c) utilize the labeled information. Here cluster centers and feature combinations are encoded in the form of a string. Assignment of points to different clusters is done with the use of available set of features based on the point symmetry based distance. Four objective functions, one measuring the total compactness of the partitioning based on the Euclidean distance, the second one measuring the total symmetry of the clusters, third one measuring the similarity between the available labeled information and obtained partitioning, and the last one counting the number of features, are considered here. These objective functions are optimized simultaneously using the search capability of AMOSA, a newly developed simulated annealing based multiobjective optimization method, in order to determine the appropriate feature combinations, appropriate number of clusters as well as the appropriate partitioning. The performance of the proposed algorithm named Semi-FeaClusMOO is compared with the Euclidean distance based version of the same algorithm, FeaClusMOO technique which tackles feature selection problem under unsupervised classification framework, a multiobjective based automatic clustering technique, VAMOSA, a single objective clustering technique, VGAPS, and one traditional clustering technique, *K*-means clustering, for several data sets having different characteristics. Results reveal that the proposed semi-supervised feature selection technique is capable to detect the appropriate feature combinations and appropriate partitioning from data sets having the point symmetric clusters.

In future we would like to explore some more objective functions. We would like to test our approach more extensively. In order to select a single solution from the final Pareto optimal front we have developed a semi-supervised approach. In future we would like to develop some more techniques for this purpose.
